# Reduced GSH Acts as a Metabolic Cue of OPDA Signaling in Coregulating Photosynthesis and Defense Activation under Stress

**DOI:** 10.3390/plants12213745

**Published:** 2023-11-01

**Authors:** Ashna Adhikari, Sang-Wook Park

**Affiliations:** Department of Entomology and Plant Pathology, Auburn University, Auburn, AL 36849, USA; aza0265@auburn.edu

**Keywords:** S-glutathionylation, sulfur assimilation, redox signaling, retrograde signaling, growth and defense coordination

## Abstract

12-oxo-phytodienoic acid (OPDA) is a primary precursor of jasmonates, able to trigger autonomous signaling cascades that activate and fine-tune plant defense responses, as well as growth and development. However, its mechanism of actions remains largely elusive. Here we describe a dual-function messenger of OPDA signaling, reduced glutathione (GSH), that cross-regulates photosynthesis machinery and stress protection/adaptation in concert, optimizing plant plasticity and survival potential. Under stress conditions, the rapid induction of OPDA production stimulates GSH accumulation in the chloroplasts, and in turn leads to protein S-glutathionylation in modulating the structure and function of redox-sensitive enzymes such as 2-cysteine (Cys) peroxiredoxin A (2CPA), a recycler in the water–water cycle. GSH exchanges thiol-disulfides with the resolving Cys^R^_175_, while donating an electron (e^−^, H^+^) to the peroxidatic Cys^P^_53_, of 2CPA, which revives its reductase activity and fosters peroxide detoxification in photosynthesis. The electron flow protects photosynthetic processes (decreased total non-photochemical quenching, NPQ_(T)_) and maintains its efficiency (increased photosystem II quantum yield, Φ_II_). On the other hand, GSH also prompts retrograde signaling from the chloroplasts to the nucleus in adjusting OPDA-responsive gene expressions such as *Glutathione S-Transferase 6* (*GST6*) and *GST8*, and actuating defense responses against various ecological constraints such as salinity, excess oxidants and light, as well as mechanical wounding. We thus propose that OPDA regulates a unique metabolic switch that interfaces light and defense signaling, where it links cellular and environmental cues to a multitude of plant physiological, e.g., growth, development, recovery, and acclimation, processes.

## 1. Introduction

Oxylipins, oxygenated derivatives of fatty acids, are essential signaling molecules in diverse physiological processes in plants and animals [1]. In plants, oxylipins operate a layer of defense (adaptation), growth, and ontogenetic mechanisms, while mammalian oxylipins (eicosanoids) control intricate regulatory mechanisms in immunity, functioning as messengers in the central nervous system and participating in resolution processes following tissue injury [2,3]. Moreover, recent studies have illuminated the medicinal value of plant oxylipins, highlighting their anticancer and anti-inflammatory activities [4,5,6]. Notably, the molecular components and metabolic pathways involved in oxylipin biogenesis and signaling share common ancestry and evolutionary processes across kingdoms [1]. Hence, uncovering the modes of actions associated with oxylipins will not only advance our understanding of the phenotypic and environmental plasticity of plants, but also assist the improvement of drug development via facilitating the rational design of more potent and safer anticancer (and anti-inflammatory) drugs. However, our current knowledge regarding oxylipin signaling is still incomplete [2,3].

Recently, molecular underpinnings have been investigated for (+)-12-oxophtodienoic acid [OPDA; (1*S*,2*S*)-3-oxo-2-(2*Z*-pentenyl)-cyclopent-4-ene-1-octanoic acid], activating an autonomous signaling that regulates a unique subset of jasmonate-responsive genes [7,8,9]. OPDA is a primary precursor of the jasmonate family of plant oxylipins, derived from trienoic fatty acids via the octadecanoid pathway in the chloroplasts. Once accumulated, a portion of OPDA travels to the peroxisomes and undergoes β-oxidations to form (−)-jasmonic acid [JA; (1*R*,2*R*)-3-oxo-2-(2*Z*)-2-pentenyl-cyclopentaneacetic acid] [6]. In the meantime, the rest of the OPDA sets out metabolic cascades via binding and stimulating its receptor, cyclophilin 20-3 (CYP20-3), to form a complex with serine acetyltransferase 1 (SAT1), which stimulates the formation of hetero-oligomeric cysteine (Cys) synthase complex (CSC) with *O*-acetylserine(thiol)lyase B. CSC formation then actuates sulfur assimilation, which leads to increased levels of thiol metabolites (e.g., glutathione, GSH) and the buildup of cellular reduction potential. The enhanced redox capacity in turn coordinates the expression of a subset of OPDA-responsive genes (ORGs) in activating and calibrating disease resistances, and defense responses against various abiotic stresses [10,11].

CYP20-3 is a dual-function enzyme, exerting peptidyl-prolyl *cis*-*trans* isomerase (PPIase) and reductase activities, that directly interacts and metabolizes cofactor proteins such as SAT1 (see above) and 2-Cys peroxiredoxin A (2CPA) [10,11,12,13,14]. 2CPA is a highly abundant plastid peroxidase, protecting and modulating photosynthetic mechanisms [13]. However, 2CPA typically forms an obligatory homodimer as the peroxidatic Cys (C^P^)_53_ from one monomer is connected via a disulfide bond to the resolving Cys (C^R^)_175_ located at another monomer. The oxidation of C^P^_53_, in consequence, arrests the catalytic activity of 2CPA. Therefore, 2CPA dimers require electron (e^−^, H^+^) donors such as thioredoxins (TRXs), NADPH-dependent TRX reductase C, and/or CYP20-3, which reduces (activates) it to be able to metabolize the detoxification of a toxic byproduct (i.e., H_2_O_2_) in photosynthesis and the operation of fructose 1,6-bisphosphatase, a key enzyme in the Calvin cycle [12,13,15,16,17]. Hence, deficiency of 2CPA in antisense and T-DNA insertion mutant plants manifested the increased levels of H_2_O_2_ and carbonylated proteins, while decreasing the quantum yield of photosystem II (PSII) and CO_2_ fixation rates, which together result in growth and developmental inhibition [18,19,20,21]. Reduction of 2CPA thereby enhances its antioxidant capacity and fosters photosynthetic efficacy, ensuring optimal growth, reproduction, and survival of plants [11,12,20,22].

GSH is the most prevalent nonprotein thiol in plants, playing a crucial role in maintaining cellular redox homeostasis under different ecological conditions. Most important-ly, it reduces reactive oxygen species (ROS) and other peroxides by providing its electron (e^−^, H^+^), and is subsequently oxidized to a disulfide form (GSSG). Hence, increased levels in cellular GSH and attendant reduction capacity often coincide with the generation of free radicals and oxidants, supporting a notion that GSH status is involved in transmitting oxidative stress signaling [23]. The oxidative bursts, therefore, decrease GSH:GSSG ratios, which in turn stimulates the reversible formation of mixed disulfides between protein sulfhydryl groups (PSH) and GSSG (i.e., S-glutathionylation). S-glutathionylation then engenders the structural and functional modification of redox-sensitive enzymes, reformatting the electron transport chain (ETC) of cellular metabolic and signaling pathways in plant growth, immune responses, and injury recovery [24]. For instance, under ROS stresses, 2CPA was originally believed to be hyperoxidized and become homodecamers that lose their peroxidase activities, but instead gain chaperone activity against oxidative damage [15]. However, later studies with human PrxI and pea 2CP detailed that they are S-glutathionylated by GSSG, shifting their quaternary structures back from decamers to dimers, and subsequently inactivating their molecular chaperone activity, proposing the alternative roles of 2CPA via S-glutathionylation in response to cell signaling and oxidative stress [25,26,27].

In the present study, we unveil the unique mode and activity of S-glutathionylation that conveys plant defense, OPDA, hormone signaling to modulate ROS levels and photosynthetic rates during plant defense and repairing processes against various environmental stresses. Mechanistically, under stress conditions, CYP20-3-dependent OPDA signaling induces high levels of GSH which triggers the S-glutathionylation (posttranslational activation) of 2CPA in accelerating peroxide detoxification and maintaining energy supply, whilst actuating retrograde (from plastids to nucleus) signaling that coordinates ORG expression and plant defense activation against various exterior pressures. The mode of OPDA signaling thus reconstitutes GSH as a unique and independent signal cue in controlling the interplay between growth and defense responses, which makes instant and appropriate adaptive decisions to maximize plant plasticity and survival (‘fitness’) under a range of environmental constraints.

## 2. Results

### 2.1. GSH Binds and Determines the Quaternary Structure of 2CPA in the Chloroplasts

**Figure 1:**
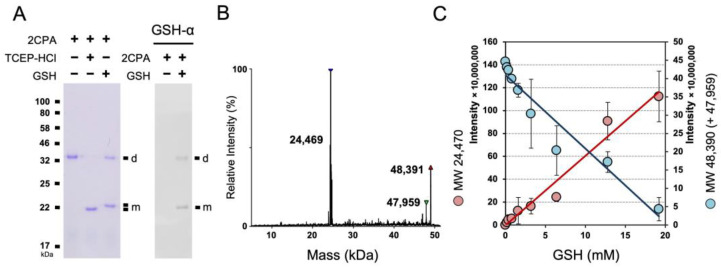
GSH-dependent S-glutathionylation determines the cellular structure and function of 2CPA. (**A**) Reduced GSH S-glutathionylates and monomerizes 2CPA. The 1 µM 2CPA, incubated with/without 1 mM GSH or TCEP-HCl (a reducing agent) for 30 min, was subjected to nonreducing SDS/PAGE (left panel) and immunoblot (IB, right panel), analyzed using monoclonal anti-GSH antibody (GSH-α, noted on the top). (**B**,**C**) LC/MS analysis of GSH-treated 2CPA. A representative spectra (**B**) and the kinetics (**C**) of deconvoluted masses of 2CPA incubated with increased concentrations (0, 0.2, 0.4, 0.8, 1.6, 3.2, 6.4, 12.8, and 19.2 mM) of GSH and 15 mM NEM. Three major peaks in (**B**) indicate a monomeric 2CPA^GS^ (~24.409 kDa = 23.957_2CPA_ + 0.125_NEM_ + 0.307_GSH_), a dimeric 2CPA^GS^ (~48.391 kDa = 2× 23.957_2CPA_ + 0.125_NEM_ + 0.307_GSH_), and a dimeric 2CPA (~47.959 kDa = 2× 23.957_2CPA_). d, dimeric 2CPA. m, monomeric 2CPA. Note that all proteins used throughout this study were tag-free versions.

To further delineate the OPDA signaling circuitry, we investigated the cellular and organismal activity of an OPDA receptor, CYP20-3, and its cofactors, including 2CPA [11,14,28]. 2CPA, however, appeared to bind a reduced form of GSH, and became insensitive to the enzymatic (reductase) activity of CYP20-3, unless conditioned at high temperatures (≥36 °C) [28]. Thus, we inspected if and/or how 2CPA mechanistically binds GSH using nonreducing SDS/PAGE and LC/MS separations (Figure 1 and Figure S1A). Both analyses indeed corroborated that increases in GSH concentrations progressively promote total the S-glutathionylation of 2CPA, and subsequently lead to its monomerization. As shown in Figure S1B–D without GSH, 2CPA was obligatorily oxidized and dimerized by itself (~47.959 kDa, Table S1). GSH (0.307 kDa) supplementation then steadily targeted and S-glutathionylated 2CPAs, increasing its molecular weight (MW) to ~48.390 kDa (2× 23.975_2CPA_ + 0.125_NEM_ + 0.307_GSH_), and eventually cleaving them to monomeric species ~24.409 kDa (23.975_2CPA_ + 0.125_NEM_ + 0.307_GSH_) in a concentration-dependent manner (Figure 1B,C).

**Figure 2:**
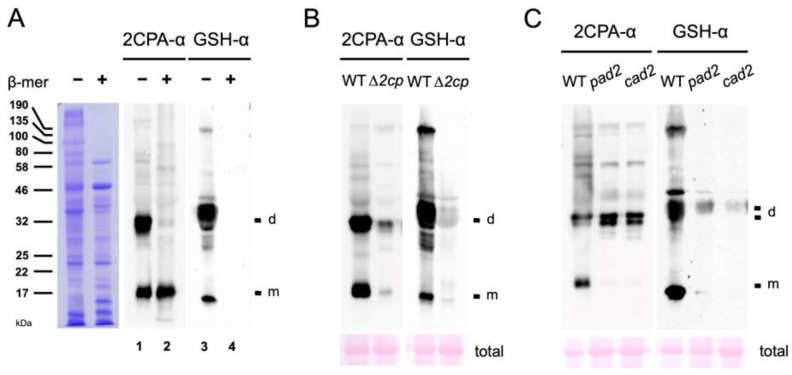
GSH-dependent S-glutathionylation determines the structure homeostasis of 2CPA in planta. (**A**) Equal amounts of total protein extracts prepared from WT (Col-0) were subjected to nonreducing (−β-mer) and denaturing (+β-mer) SDS/PAGE (left panel), and IB analyzed by polyclonal anti-2CPA antibody (2CPA-α, middle panel) and GSH-α (right panel). (**B**,**C**) Equal amounts of total proteins extracted from WT (Col-0; **B**,**C**) and *∆2cp* (*2cpa* and *2cpb* double mutant) (**B**) or *pad2* and *cad2* (GSH-deficient mutants) (**C**) were separated in nonreducing SDS/PAGE and IB analyzed by 2CPA-α (left panel) and GSH-α (right panel). PVDF membranes of total Arabidopsis extracts were stained with Ponceau S (bottom). d, dimeric 2CPA. m, monomeric 2CPA.

The in vitro results (Figure 1A and Figure S1A) indicate that the basal-level cellular concentrations of GSH in the chloroplasts (~1 mM) [29] where 2CPA is localized are sufficient to S-glutathionylate part of 2CPAs in plants. To substantiate this hypothesis, we compared the MW of 2CPA with those of GSH-bound proteins in total Arabidopsis extracts using anti-2CPA (2CPA-α) and anti-GSH (GSH-α) antibodies (Figure 2A). In the nonreducing condition (-β-mer), both antibodies cross-reacted with several proteins, including two major bands (lanes 1 and 3) that correspond to monomeric and homodimeric 2CPAs, respectively, as those were markedly attenuated in T-DNA insertion mutants of *2CPs* (*∆2cp*, Figure 2B). Note that *∆2cp* is not a complete null mutant of *2CPA*, displaying residual-level accumulations of its mRNA and protein (Figure 2B and Figure S1E), as previously described [20]. In comparison, the supplement of reductant (+β-mer) engendered the monomerization of 2CPA (Figure 2A, lane 2) and the de-glutathionylation of all GSH-binding proteins (lane 4), reassuring that S-glutathionylation, induced by thiol-disulfide exchanges, defines the conformation. In further support, the depletion of GSH accumulations in *pad2* and *cad2* mutants [30,31] notably decreased 2CPA S-glutathionylation (Figure 2C, right panel) and, as a result, hindered monomerizing 2CPA (left panel). The need for GSH-producing enzyme, *γ*-glutamylcysteine synthetase, in 2CPA S-glutathionylation (Figure 2C) clearly defines the intrinsic role of GSH in balancing the redox and structural homeostasis of 2CPA upon arriving in the chloroplasts.

### 2.2. GSH-Dependent S-Glutathionylation Stimulates the Enzymatic Activity of 2CPA

**Figure 3:**
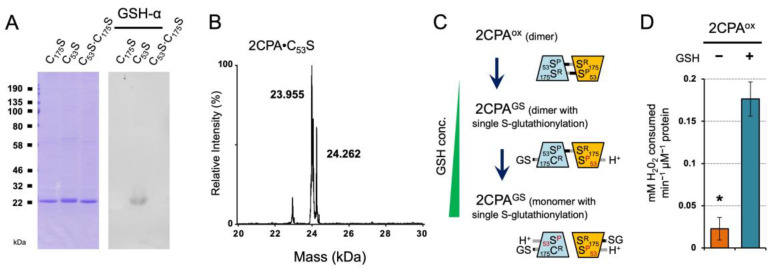
GSH-dependent S-glutathionylation determines the cellular structure and function of 2CPA. (**A**,**B**) Reduced GSH binds only the Cys53 to Ser mutagenized 2CPA (C_53_S). Three single or double Cys to Ser mutant 2CPAs (C_53_S, C_175_S, and C_53_S·C_175_S, 1 µM) were incubated with 2 mM GSH for 30 min, and IB analyzed by GSH-α (**A**) and separated in LC/MS (B and Figure S1F). Two major peaks in (**B**) indicate a monomeric 2CPA·C_53_S (~23.955 kDa) and 2CPA·C_53_S^GS^ (~24.260 kDa = 23.955_2CPA·C175S_ + 0.307_GSH_). (**C**) Proposed mode of GSH-dependent S-glutathionylation of 2CPA. From the top, GSH exchanges S-S bond(s) with the C^R^_175_ residue of 2CPA^ox^ dimer, while donating electron (H^+^, e^−^) to the adjacent C^P^_53_ residue, which releases single S-glutathionylated 2CPA^GS^ dimer (middle) and monomer (bottom) in a GSH concentration-dependent manner. GSH conc., GSH concentrations. (**D**) S-glutathionylation activates 2CPA^ox^. Peroxidase activity was measured in 2CPA^ox^ and 2CPA^GS^ by incubating with 50 µM H_2_O_2_ for 10 min. H_2_O_2_ was then quantified using the eFOX method [32] (mean ± SD; *n* = 3). The asterisk (*) indicates statistically significant differences (*p* < 0.05, the student’s *t*-test). Note that all proteins used throughout this study were tag-free versions.

The LC/MS analysis identified that GSH-binding yields dimeric and monomeric 2CPA species through the single S-glutathionylation (~24.409 and ~48.390 kDa, Figure 1B and Table S1). To understand the functional context for the S-glutathionylation, we assessed if GSH targets a specific or random Cys between two C^P^_53_ and C^R^_175_ residues of 2CPA, by probing the binding capacity of GSH to three different Cys to Serine (Ser) single- or double-mutagenized 2CPA (C_53_S, C_175_S and C_53_S∙C_175_S) proteins (~23.955 kDa, Figure 3A,B and Figure S1F). The immunoblot (IB) detection revealed that GSH binds only 2CPA∙C_53_S (Figure 3A) and releases an extra species with increased MW ~24.260 kDa (23.955_2CPA∙C53S_ + 0.307_GSH_, Figure 3B), elucidating that GSH selectively S-glutathionylates the C^R^_175_, and its occurrence with one or both C^R^_175_ from 2CPA^ox^ dimers determines the quaternary structure (e.g., dimer and monomer) of S-glutathionylated 2CPA^(GS)^ (Figure 3C). On the other hand, the catalytic Cys (C^P^_53_) thus becomes available to receive an electron (e^−^, H^+^) from GSH and in turn activates the peroxidase activity of 2CPA^GS^, enabling the reduction and detoxification of H_2_O_2_ (Figure 3D). The result is in agreement with the conclusion that GSH acts as an electron (e^−^, H^+^) donor to 2CPA.

### 2.3. GSSG-Dependent S-Glutathionylation Protects 2CPA against Oxidative Stresses

**Figure 4:**
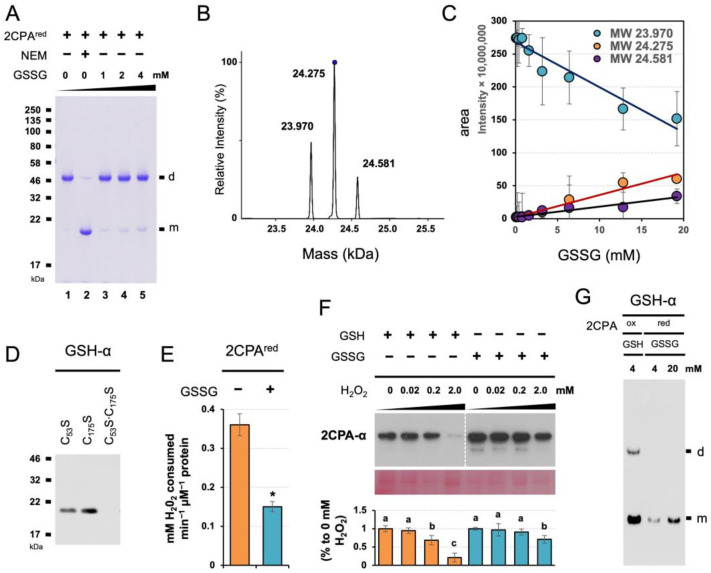
GSSG-dependent S-glutathionylation deactivates but protects 2CPA from hyperoxidative denaturation. (**A**) GSSG binds and prevents 2CPA^red^ from its oxidation and dimerization. The 1 µM reduced 2CPAs^(red)^, incubated with/without various concentrations (0, 1, 2 and 4 mM) of GSSG or 30 mM NEM for 30 min, was subjected to nonreducing SDS/PAGE. (**B**–**D**) GSSG leads double S-glutathionylation at the Cys53 and Cys175 residues of 2CPA^red^. Representative spectra (**B**) and the kinetics (**C**) of deconvoluted masses of 2CPA^red^ incubated with increased concentration (0, 0.2, 0.4, 0.8, 1.6, 3.2, 6.4, 12.8, and 19.2 mM) of GSSG. Three major peaks in (**B**) indicate a monomeric 2CPA without (~23.970 kDa) and with single (~24.275 kDa = 23.970_2CPA_ + 0.307_GSH_) or double (~24.581 kDa = 23.970_2CPA_ + 2× 0.307_GSH_) S-glutathionylation. (**D**) Mutant 2CPAs (C_53_S, C_175_S, and C_53_S·C_175_S) were analyzed by IB using GSH-α, following 30 min incubation with 20 mM GSSG. (**E**) GSSG binding inactivates 2CPA^red^. Peroxidase activity was measured using eFOX method [32] in 2CPA^red^ and 2CPA^GS^ with double S-glutathionylation by incubating with H_2_O_2_ for 10 min (mean ± SD; *n* = 3). The asterisk (*) indicates statistically significant differences (*p* < 0.05, the student’s *t*-test). (**F**) GSSG-mediated S-glutathionylation suppresses H_2_O_2_-induced deactivation of 2CPA. Equal amounts of total plastid proteins were pretreated overnight with 2 mM of GSH or 20 mM of GSSG, and dialyzed by desalt columns. The plastid proteins^GS^ were then incubated with the indicated concentration of H_2_O_2_ for another 30 min at 37 °C, and subjected to denaturing SDS/PAGE and IB analyzed using 2CPA-α. The PVDF membrane of chloroplast protein extracts was stained with Ponceau S (bottom). The level IB signals of 2CPA were quantified (mean ± SD; *n* = 3) through Image J v1.53 [37], and normalized to the expression of PSII monomer. Different letters indicate significant differences between H_2_O_2_ concentrations (Tukey–Kramer honestly significant difference test on all pairs; α = 0.05). (**G**) GSH binds 2CPA^ox^ more strongly than GSSG toward 2PCA^red^. The 1 µM 2CPAs^red^ or 2CPAs^ox^, incubated with various 4 mM GSH or 4 and 20 mM of GSSG, respectively, was separated in nonreducing SDS/PAGE and IB analyzed using GSH-α. d, dimeric 2CPA. m, monomeric 2CPA. Note that all proteins used throughout this study were tag-free versions.

The unique mode and cellular activity of GSH-dependent 2CPA S-glutathionylation prompted us to assess if and/or how GSSG S-glutathionylates 2CPA, once examined in a pea using a reduced form (pretreated with 10 mM DTT) of Ps2CPA^(red)^ [27]. In running the nonreducing SDS/PAGE (Figure 4A), 2CPA^red^ monomers (visible when their free thiols were alkylated/blocked by NEM, lane 2) were mostly oxidized to dimers (lane 1) through either strong oxidants used as initiators in gel polymerization such as ammonium persulfate or molecular oxygen generated during the gel polymerization [33]. In this condition, GSSG supplementations progressively prevented the artifactual oxidation of 2CPA^red^ in a concentration-dependent manner (lanes 3 to 5), indicating that GSSG forms mixed disulfides with the PSH of 2CPA^red^ and, as a consequence, occludes 2CPA dimerization. GSSG could target, unlike GSH, both C^P^_53_ and C^R^_175_ of 2CPA^red^ (Figure 4B) and formed double S-glutathionylation ~24.581 kDa (23.975_2CPA_ + 2x 0.307_GSH_, Figure 4C,D). S-glutathionylation of the C^P^_53_ (catalytic Cys) then led to the deactivation of 2CPA^red^ peroxidase activity (Figure 4E vs. Figure 3D), illuminating the potentially different role of GSSG vs. GSH in controlling the cellular activity of 2CPA. Note that GSSG did not S-glutathionylate 2CPA^ox^ (Figure S1G,H). In this respect, protein S-glutathionylation by GSSG is often considered a way to protect irreversible oxidation of, while inactivating, protein thiols, and the proteins^GS^ are reactivated/restored by deglutathionylating enzymes such as sulfiredoxin and glutaredoxin when oxidative stress conditions are over [18,19,25,27,34,35,36]. We hence surveyed the structural integrity of single (GSH-treated) and double (GSSG-treated) S-glutathionylated 2CPAs against hyperoxidation (Figure 4F). The IB analysis detecting level 2CPAs exhibited, as anticipated, that the pre-treatment of GSSG sustains 2CPA structures better than GSH against the higher concentrations (e.g., 2 mM) of H_2_O_2_. However, GSSG-induced S-glutathionylation of 2CPA^red^ was clearly less effective than GSH toward 2CPA^ox^ (Figure 4G), entailing excessive negative potentials (≥1 mM GSSG, Figure 4A,D) that are ≥10-fold greater than its cellular concentrations (~70–100 µM) [29]. The results together with the fact that 2CPA is obligatorily an oxidized dimer (Figure S1B–D) [17] reconstitute that GSH is, rather than GSSG, a preferential modifier of 2CPA S-glutathionylation. Indeed, increased GSH:GSSG ratios (>14:1) stimulate 2CPA S-glutathionylation, whereas decreased reduction capacity (≤14:1) showed little effect on 2CPA S-glutathionylation [28].

### 2.4. GSH-Dependent S-Glutathionylation Relays an OPDA Signal in Protecting Photosynthesis under Environmental Stresses

**Figure 5:**
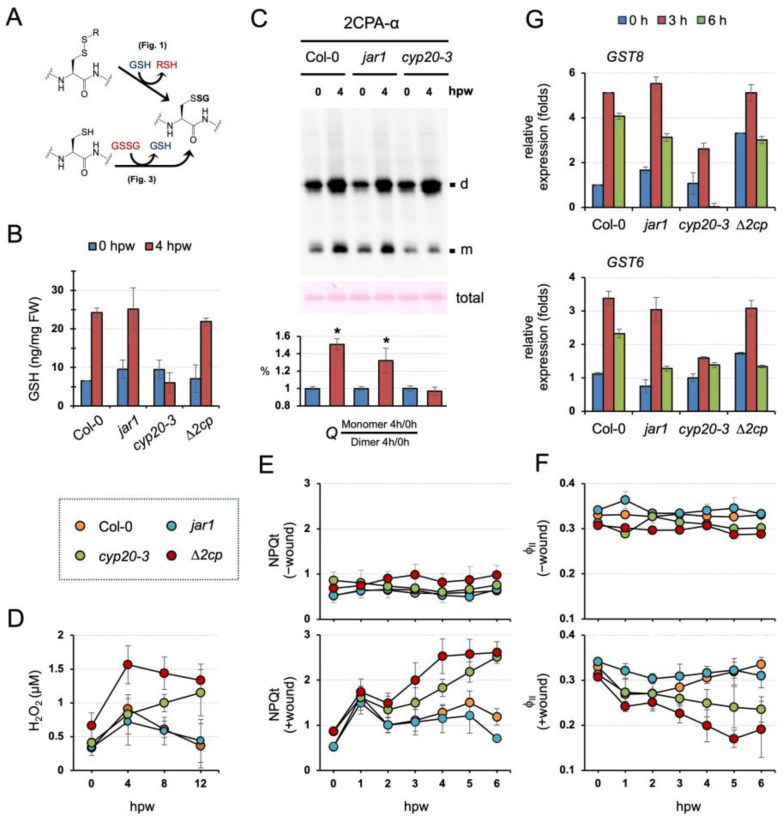
GSH-dependent 2CPA S-glutathionylation relays CYP20-3/OPDA signaling in the maintenance of photosynthesis during wound healing. (**A**) The representative schematic of GSH- or GSSG-dependent protein S-glutathionylation. (**B**) CYP20-3/OPDA signaling stimulates wound-responsive accumulations of GSH. Levels of GSH in stressed WT and mutant (*jar1*, *cyp20-3,* and *∆2cp*) plant leaves extracted at 0 and 4 hpw were measured (mean ± SD; *n* = 9) utilizing a fluorometric assay kit (BioVision), according to the manufacturer’s instruction. The results of statistical analyses are summarized in Table S2. (**C**) The disruption of CYP20-3/OPDA signaling impairs wound-responsive S-glutathionylation and monomerization of 2CPA. In situ IB assay determines the quaternary structure of 2CPA in wounded WT (Col-0), JA-Ile synthesis mutant (*jar1*), and OPDA signaling mutant (*cyp20-3*) plants at 0 and 4 hpw. The level IB signals of monomeric 2CPAs (*Q*) were quantified (mean ± SD; *n* = 3) by Image J v1.53 [37], and normalized to those of dimeric forms (bottom). The asterisk (*) indicates statistically significant differences (*p* < 0.05, the student’s *t*-test). (**D**–**F**) CYP20-3/OPDA signaling promotes the peroxidase activity of 2CPA in protecting photosynthesis under wound healing. Time-course changes of H_2_O_2_ levels (**D**) as well as NPQt (**E**) and ΦII (**F**) values in unwounded (-wound) or wounded (+wound) WT (Col-0) and mutant (*jar1*, *cyp20-3,* and *∆2cp*) plants (means ± SD; *n* = 10). The results of statistical analyses by Tukey–Kramer honestly significant difference test on all pairs are summarized in Tables S3 and S4. (**G**) Time-resolved qRT-PCR analysis of *GST8* and *GST6* in wounded WT (Col-0) and mutant (*jar1*, *cyp20-3,* and ∆*2cp*) plants. Total RNAs were prepared from leaves at 0, 3, and 6 hpw. Values were normalized to the expression of *POLYUBIQUITIN* (mean ± SD; *n* = 3) [38]. d, dimeric 2CPA. m, monomeric 2CPA.

Our data suggest two distinctive modes and activities of S-glutathionylation in conditioning the cellular structure and function of 2CPA (Figure 5A). In line with this scenario, our earlier study revealed that CYP20-3 relays an OPDA signal to stimulate GSH biogenesis, independently of the oxidative bursts, under stress, e.g., wounding (Figure 5B and Figure S2A, Table S2) [11]. These together postulate that OPDA signaling conveys stress inputs into the output, the activation of 2CPA, that maintains photosynthetic redox homeostasis [15,16,17]. To substantiate this hypothesis, we first IB surveyed ex vivo dynamics of 2CPA S-glutathionylation in conjunction with wound-responsive jasmonate biosynthesis. Wounding induced the accumulation of OPDA with a peak at ~1 to 3 h postwounding (hpw), subsequently JA-Ile, and ultimately the differential expression of various genes (Figure S2B) [11]. As anticipated, 2CPA S-glutathionylation was promoted in parallel with OPDA, but not JA-Ile, signaling, which channels wound-responsive GSH synthesis (Figure 5B,C and Figure S2, and Table S2). At 4 hpw, level 2CPAs were increasingly upregulated while progressively monomerized in WT and *jar1* (+OPDA/-JA-Ile signaling), whereas they mostly accumulated as dimers in the *cyp20-3* (-OPDA/+JA-Ile signaling), indicating that GSH-dependent S-glutathionylation conveys OPDA signaling to drive redox reaction routes that accelerate peroxide detoxification capacity (elevated 2CPA activity) during plant stress recovery/acclimation processes.

In agreement with the in situ IB results, *cyp20-3* impaired the timely removal of wound-responsive H_2_O_2_ accumulations that otherwise peaked at 4 hpw and were steadily neutralized, as shown in wounded WT and *jar1* (Figure 5D and Table S3). Wounded *∆2cp*, on the other hand, accumulated significantly higher H_2_O_2_ amounts at 4 hpw than wounded WT and *jar1*, corroborating an essential role of 2CPA in resolving stress-responsive increases in H_2_O_2_, which is functionally coupled with protecting photosynthetic machinery [17]. We hence assessed if the wound-reactive spike of H_2_O_2_ antagonizes photosynthetic efficiency. As expected, wounded *∆2cp* and *cyp20-3* reduced the utility of light energy lower than wounded WT and *jar1*, as revealed by the extensive total nonphotochemical quenching [39] (NPQ_(T)_, Figure 5E and Table S4) and the decreased photosynthetic efficiency (photosystem II quantum yield (Φ_II_) [40], Figure 5F and Table S4). These results describe that CYP20-3-dependent GSH synthesis and its protein (e.g., 2CPA) S-glutathionylation (referred to as ‘reductant (GSH) signaling’) relay an OPDA defense signal in maintaining photosynthetic processes and efficiency, shedding light on a unique regulatory hub and interplay between growth and defense processes. Indeed, the disruption of reductant signaling in *cyp20-3* also attenuated the wound-responsive upregulation of ORG expressions such *as GLUTATHIONE S-TRANSFERAE 8 (GST8*) and *GST6* [8] (Figure 5G), which activate defense responses [41]. However, wounded *∆2cp* exhibited WT-like wound responses in *GST8* and *GST6* expressions, indicating that GSH and its reductant signaling are an upstream regulator and metabolic pathway in the maintenance of photosynthesis and the activation of defense gene expressions.

### 2.5. A Programmed Synthesis of GSH by CYP20-3-Dependent OPDA Signaling Is Intrinsic in Plant Stress Defense and Acclimation

**Figure 6:**
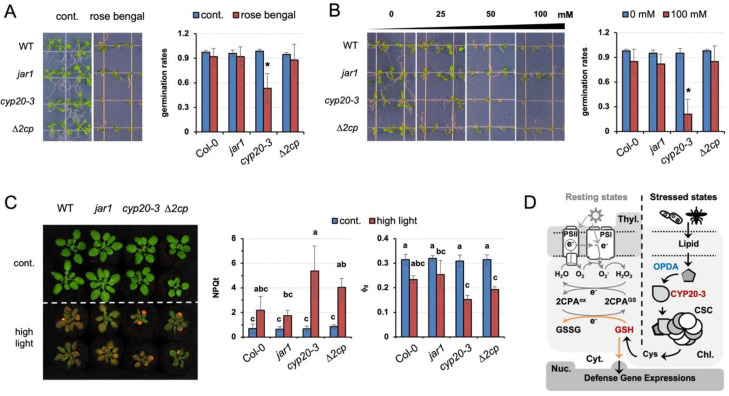
Programmed induction of GSH by OPDA signaling plays a crucial role in plant defense responses. (**A**–**C**) The OPDA signaling mutant, *cyp20-3*, impairing stress-responsive GSH induction (Figure 5B), is hypersensitive to excess ROS stresses, compared to WT (Col-0) and *jar1* and *∆2cp* mutant plants. Seeds were plated on MS agar medium without or containing 3 µM rose bengal (**A**) or different concentrations (0, 25, 50, and 100 mM) of NaCl (**B**), and their germination rates were counted on the fifth day after vernalization (means ± SD; *n* = 25). (**C**) The 2-week-old plants, grown under normal conditions, were transferred to high-intensity light for 6 days, and subjected to the measurement of Φ_II_ values (means ± SD; *n* = 10). Photographs were taken on the seventh to fourteenth days after treatments. The asterisks (*) and different letters indicate statistically significant differences (Tukey–Kramer honestly significant difference test on all pairs; α = 0.05). (**D**) Proposed model of GSH-dependent reduction signaling that relays OPDA signaling to cross-regulate an interplay between photosynthesis (growth) and ORG expressions (defense) under stressed conditions. When the PSI antenna captures solar energy (in resting states), it prompts the ETC that controls energy (sugar) conversion and consumption. By contrast, under stressed conditions, OPDA is accumulated and binds CYP20-3 to stimulate the formation of CSC (Cys synthase complex) and the generation of Cys and GSH, which in turn coordinates (i) the S-glutathionylation (activation) of 2CPA in peroxide detoxification, while (ii) triggering the retrograde regulation of defense ORG expressions. This regulatory interface between growth and defense responses shapes the optimal growth plasticity and survival potential of plants under constant environmental pressures.

Our results explain the role of GSH in relaying CYP20-3-dependent OPDA signaling, which delivers different stress cues to two distinct cellular processes, (1) fostering 2CPA activations in photosynthesis and (2) coordinating ORG expressions. The latter then reprograms plant cells toward defense modes against various environmental challenges [9]. Thus, *cyp20-3*, impeding stress-responsive GSH biogenesis (Figure 5B, Figures S2A and S3, and Table S2) [11], manifested hypersensitivity to various exterior stresses, including rose bengal, a light-dependent inducer of ROS, singlet oxygen (^1^O_2_, Figure 6A). When seeds were plated on Murashige and Skoog (MS) medium supplemented with 3 µM rose bengal, germination and growth of *cyp20-3* seedlings were significantly suppressed relative to WT, *jar1,* and *∆2cp*, validating a crucial role of OPDA-induced reductant signaling in stress and defense responses.

Abiotic stresses such as elevated salt levels and high light also damage plants by producing ROS. When subjected to these stresses, *cyp20-3* showed a response similar to that observed with rose bengal (Figure 6B,C). Under the increasing concentrations of excess NaCl (25 to 100 mM), the germination rate of *cyp20-3* seedlings was progressively inhibited (Figure 6B). Similarly, high light treatment (300 µmol photons m^−2^s^−1^) severely inhibited growth and photosynthetic efficiency in all plants (Figure 6C). Once again, however, *cyp20-3* suffered the most, whereas *jar1* (-JA-Ile signaling) grew noticeably larger in the optimal conditions and exhibited a slight increase in photosynthesis under high light intensity. These results together allow us to locate CYP20-3 and its GSH production at a central metabolic pathway of OPDA signaling which cross-regulates light-dependent ETC (growth) and ORG expressions (defense) under stressed states (Figure 6D).

## 3. Discussion

In nature, plants must grow and defend themselves to survive and reproduce. A caveat is that defense activations come at the expense of growth and vice versa [42]. This phenomenon, referred to as ‘growth and defense tradeoff’, effectively circumstantiates plant responses toward the persistent and/or excess surges of environmental pressures. Plants, however, are more often situated in resisting a series of transient and modest-level stresses, while concurrently completing growth to achieve maximum yields and development. Thus, recent studies have begun to elaborate an alternative model, ‘growth and defense coordination’, wherein a balancing act between growth and defense can collectively optimize plant fitness and survival [43]. For instance, perception of changes in light intensity leads to the spatial production of auxins, which fosters phototropic growth and, at the same time, initiates jasmonate signaling, which actuates defense machinery in Arabidopsis [44,45,46]. Auxins, however, suppress JA-Ile signaling via promoting gibberellin (GA) synthesis, which in turn triggers the degradation of DELLA proteins, negative regulators of GA but activators of MYC2 TF [46,47,48]. MYC2 is a key TF relaying COI1/JA-Ile signaling [49]. Hence, the light-responsive jasmonate signaling is likely conveyed by OPDA, another biologically active jasmonate, which is in fact induced by EL intensity on a time scale of hours, with the concomitant accumulation of a subset of jasmonate-responsive genes [50,51]. In this context, the disruption of OPDA signaling (*cyp20-3*) manifested both plant growth retardation under EL, and hypersensitivity toward EL-originated oxidative stress (Figure 6A,C) [10]. Furthermore, a sorghum inbred line accumulating higher-level OPDA displayed not only enhanced defense capacity to aphid attacks, but also minimal biomass loss as well as a robust photosynthetic machinery. On the other hand, the aphid-tolerant line accumulated similar or attenuated levels of JA-Ile and other plant defense-associated hormones, including salicylic acid (SA) and cytokinin, comparing to aphid-susceptible lines [52]. The results from our research and that of other groups indicate a crucial role of OPDA signaling as a key facet of plant growth and defense coordination, which assists in making instant and appropriate adaptive decisions to maximize plant plasticity and survival (‘fitness’) under a range of environmental constraints.

In the present study, OPDA activates CYP20-3-dependent GSH biogenesis, which deploys redox-dependent signal transmissions in (i) stimulating the retrograde regulation of defense ORG expressions, while (ii) enriching the S-glutathionylation (activation) of 2CPA, which optimizes photosynthetic efficacy and redox homeostasis (Figure 6D). In light of this, 2CPA S-glutathionylation appears to convey two distinctive kinds of metabolic, cellular redox signaling. Thus far, a number of studies have focused on the activity of GSSG-dependent 2CPA^red^ S-glutathionylation in oxidative stress signaling [23,25,35]. When GSSG levels are increased by mitigating the ROS burst under environmental stresses, GSSG targets and forms disulfide bonds with two PSHs at C^P^_53_ and C^R^_175_ of 2CPA^red^, resulting in its deactivation but protection against ROS damage (e.g., Figure 4B–F). Hence, GSSG-dependent 2CPA^red^ S-glutathionylation is proposed to relay ‘oxidant signaling’ in fostering organismal adaptations to new environmental changes [25,53,54]. However, the GSSG-dependent PTM often requires ~10-to-50-fold higher GSSG concentrations than its cellular levels, and 2CPA is found to obligatorily form an oxidized homodimer [17,23,27,29,55,56], needing further validation of its physiological efficacy and relevance. Herein, we observed that GSH could be more effective than GSSG (>~8-folds, Figure 4G), at its physiological concentrations, in S-glutathionylating 2CPA^ox^ (Figure 1 and Figure 2). Interestingly, GSH confers differential structural and functional PTM, compared with GSSG (Figure 3 and Figure 4). GSH targets only one PSH (C^R^_175_, non-catalytic thiol) and, instead, reduces catalytic thiol (C^P^_53_), switching on the activation of oxidized 2CPA dimer to monomer, and maintaining optimal photosynthesis.

In plants, photosynthesis, transforming sunlight and water into chemical energy that fuels growth and survival under various ecological conditions, is, on the other hand, a principal manufacturer of H_2_O_2_. In the light-dependent reactions, a water molecule is split to O_2_ as a high-energy waste product at PSII. Subsequently, the reduction of O_2_ at PSI generates superoxide radical (O^−^_2_) and releases H_2_O_2_, which in turn reduces photosynthetic activity to half and stimulates the production of hydroxyl radicals (•OH), causing irreversible damage in the chloroplasts [57]. Hence, H_2_O_2_ in optimal conditions is rapidly scavenged back to water by ascorbate peroxidases (called the water–water cycle, WWC), safely dissipating excess excitation energy. When exposed to stresses, however, most ascorbate peroxidase (APX) isoforms, highly sensitive to oxidative inactivation, become completely deactivated [58], entailing different peroxidases to complete the O_2_-dependent sequential reactions. In line with this scenario, we revealed that (i) arresting the peroxidase activity of 2CPA manifests drastic increases in H_2_O_2_ concentrations, and attendant decreases in photosynthetic efficiency, under stress (Figure 5D–F and Tables S3 and S4), and (ii) the 2CPA-dependent, APX-independent, stimulation of WWC is intertwined with a major plant defense apparatus actuating OPDA signaling, which in turn stimulates reductant signaling (GSH-dependent S-glutathionylation) and peroxidase activity of 2CPA (Figure 3D, Figure 5B,C, and Figure S2, and Table S2). These results further validate a previous hypothesis that 2CPA is a critical H_2_O_2_ scavenger in preventing photoinhibition from occurring under environmental pressures, safeguarding and enriching a flux of linear electron flow in the photosynthesis step [22].

Besides its role in photosynthesis, the OPDA/GSH pathway puts forward an alternative, retrograde directional route of reductant signaling, which sheds new insights on regulatory gaps in plant defense hormone (e.g., OPDA and SA) signaling. Under stressed conditions, OPDA and SA accumulated in the chloroplasts and/or cytosol promote GSH accumulations and build up cellular reduction capacity, which in turn coordinate defense gene expressions [8,11,53,59]. In these signaling pathways, GRX480 can be considered as a multifunctional transcriptional regulator (TR), as it can be induced by both OPDA and SA. GRX480 is an electron carrier using GSH as a cofactor that binds and regulates a series of TGA TFs [8,60,61]. TGAs belong to a basic leucine zipper TF family that conveys various signaling pathways involving hormones, reactive electrophilic species (RES), and ROS, in supporting environmental plasticity in plants [8,60,62]. The latter could explain signal redundancy between CYP20-3-dependent OPDA signaling and 2CPA-mediated ETC (Figure S4A). In this study, we observed that both *cyp20-3* and *∆2cp* impair the transcriptional induction of selective ORGs, particularly those involved in general defense responses such as *HEAT SHOCK PROTEIN 70.6* (*HSP70.6*), *HSP17.6,* and *GLUTAREDOXIN 480* (*GRX480*) [11,59,63]. When stressed, *cyp20-3* and *∆2cp* both cause the dysregulation of redox states, e.g., increased H_2_O_2_ accumulations (Figure S4B and Table S5), which subsequently actuate general stress tolerance (*HSPs*, Figure S4A) machineries through ROS-responsive NAC TFs [62,64]. Alternatively, stress-responsive breakage of linolenic acid in the chloroplast membranes produces RES and/or ROS in parallel to OPDA, which could regulate TGA TF-dependent transcriptions, including *GRX480* and *HSP70.6* [8]. Indeed, we were able to show in this study that oxidative ROS (e.g., GSSG) signaling shares target (e.g., 2CPA) and starting signal (e.g., GSH) metabolites with reductant signaling, which regulates general and/or signal-specific defense responses and gene expressions. 

GSH is a major redox homeostatic buffer in aerobic life, reducing oxidative damage and transmitting oxidative stress signaling throughout a range of plant growth and survival processes. However, our knowledge of mechanistic details on its function remains to be fully explained [23]. In this study, we reveal the unique role of GSH as a key, autonomous signal messenger that shapes plant plasticity and optimal phenotype (“fitness”). This model sheds new light on the understudied (i) mode of capacity of plants, being able to enhance stress responses without growth penalties, (ii) unique interface between light and hormone signaling, which fine-tune energy (e^−^, H^+^) allocations between growth and defense while being challenged constantly by environmental pressures, ultimately maximizing survival and yield potential, and (iii) signaling circuitry of OPDA and its crosstalk with other hormones and reactive species, which activate unique and conserved (general) defense mechanisms to coordinate ultimate recovery systems. Hence, future study on the finer, global aspects of S-glutathionylation and reductant signaling will further delineate the regulatory dynamics of balancing acts in plant growth and defense coordination.

## 4. Materials and Methods

### 4.1. Plant Growth Condition

Arabidopsis WT and mutants (i.e., *∆2cp*, *pad2*, *cad2*, *cyp20-3*, and *jar1*) [10,20,30,31,65] in Columbia (Col-0) background were grown in a chamber (Caron) with a 12 h light:dark cycle (80–100 µE/m^2^/s) at 22 °C and 60% to 80% relative humidity.

### 4.2. Preparation of Recombinant Proteins

The coding sequence of mature 2CPA, removing the N-terminus signal peptide of 66 amino acids, was cloned into pET28a (Novagen, Madison, WI, USA) [28] and site-specific mutations were introduced using the QuickChange II site-directed mutagenesis kit (Agilent, Santa Clara, CA, USA) and mutagenic primer sets (Table S6), according to the manufacturer’s instructions. Each recombinant WT and mutant (C_53_S, C_175_S, and C_53_S·C_175_S) protein was then expressed by 0.1 M isopropyl ß-D-1-thiogalactopyranoside in *E. coli* BL21 (DE3) and purified through a nikel- (Pur^TM^ Ni-NTA, Thermo Scientific, Waltham, MA, USA) column, as previously performed [14,28]. To remove the His-tag, purified recombinant proteins were incubated with biotinylated thrombin (U/0.5mg) in 20 mM Tris-HCl, pH 8.4, containing 150 mM NaCl and 2.5 mM CaCl_2_, which were later removed using streptavidin agarose beads (Novagen, Madison, WI, USA).

In each preparation for PAGE and LC/MS assays, 2CPAs were oxidized by treating 0.1 mM H_2_O_2_ for 15 min or reduced by applying 5 mM tris(2-carboxyethyl)phosphine hydrochloride (TCEP-HCl) for 60 min. Excess H_2_O_2_ was removed using size-exclusion chromatography (Sephadex G-25 medium, GE Healthcare), prewashed with 50 mM Tris-HCl, pH 7.5, while excess TCEP-HCl was removed by the EZ-Desalt^TM^ spin column (BioVision, Waltham, MA, USA) pretreated with deionized water, and then 10 mg/mL catalase followed by 50 mM Tris-HCl, pH 7.5, containing 0.1 mM diethylenetriamine-pentaacetic aicd. Tris-HCl was pretreated with 10 µg/mL catalase, which was removed by passage through a Microsep^TM^ centrifugal device 10K filter (Pall Corp., Port Washington, NY, USA). Finally, protein concentrations were measured using the Amresco Bradford assay kit with bovine serum albumin (BSA) as standard, and stored at 4 °C until use.

S-glutathionylation reaction of 2CPAs was typically conducted by incubating 1 or 2 µM 2CPAs with 1.0–2.0 mM GSH or GSSG in 50 mM Tris buffer, pH 7.5, at 25 °C for 30 min, while some reactions varied in GSH or GSSG concentrations between 0.5 and 20 mM. 

### 4.3. LC/MS Analysis of 2CPAs

Samples were prepared in 50 mM Tris buffer, pH 7.5, at 25 °C by treating reduced or oxidized 2CPAs with varying GSH or GSSG concentrations and/or 30 mM *N*-ehylmaleimide (NEM) for >30 min to block remaining thiols. The 2CPAs were then separated by an UltiMate 3000 UHPLC system (Thermo Scientific, Waltham, MA, USA) equipped with a Aeris C4 column (3.6 µm, 250 × 4.6 mm; Phenomenex), through an acetonitrile gradient from 90% (*v*/*v*) solvent A (0.1% [*v*/*v*] HCOOH in water) and 10% (*v*/*v*) solvent B (0.1% [*v*/*v*] HCOOH in MeCN) to 80% solvent B over 5 min at a flow rate of 0.2 mL/min [34]. Subsequently, mass spectra for all charge states were acquired using the Orbitrap Exploris mass spectrometer (Thermo Scientific, Waltham, MA, USA) between *m*/*z* 400 and 2000 in positive mode, averaged over the full-length of each protein peak, and deconvoluted to yield the molecular masses and relative intensities using ProMass for Xcalibur (Novatia, Bucks County, PA, USA). The masses used to identify glutathionylated species are given in Table S1. 

### 4.4. Peroxidase Activity Assay

Reduction of H_2_O_2_ by 2CPAs was quantified via the eFOX assay method, as described previously [32]. Briefly, the assay was performed at 37 °C in 50 mM Tris buffer, pH 7.5, containing 50 mM NaCl with 5 µM 2CPAs. Each reaction was initiated by the addition of 50 µM H_2_O_2_, then incubated for 10 min, and terminated by 2% (*w*/*v*) TCA. A volume of 500 µL eFOX reagent (250 µM Fe(NH_4_)_2_(SO_4_)_2_, 100 µM sorbitol, 100 µM xylenol orange, and 1% [*v*/*v*] in 20 mM H_2_SO_4_) was then mixed with 100 µL of the reaction solution and the reduction in H_2_O_2_ levels was tracked spectrophotometrically by measuring the difference in absorbance between 550 and 800 nm. 

### 4.5. Protein Extractions

Total protein extracts were prepared by immersing and grinding leaf tissues in liquid N_2_ to powder using a mortar and pestle. At 4 °C, ground tissues were dissolved into two volumes of 50 mM Tris buffer, pH 7.5, containing protease inhibitor cocktails (Sigma-Aldrich, St. Louis, MO, USA), agitated for 60 min, and centrifuged for 30 min at 10,000× *g*. The supernatant was collected, and immediately used for Bradford assays and immunoblot analyses.

Total chloroplast proteins were extracted by blundering leaf tissues in 1× chloroplast isolation buffer (CIB, 0.1 M Tris buffer, pH 7.8, containing 0.33 M sorbitol, 5 mM MgCl_2_, 10 mM NaCl and 2 mM EDTA) and filtered through 6 layers of muslin cloth. The collected aqueous solution was then centrifuged for 3 min at 200× *g*, and the supernatant was transferred to a new, pre-chilled tube and centrifuged again for 7 min at 1000× *g* to obtain a green pellet. The pellet was gently resuspended with 1× CIB containing 2 mg BSA, carefully layered on the top of 40% percoll^TM^ (in 1× CIB with BSA) and 100% percoll^TM^ (GE Healthcare, Chicago, IL, USA). After centrifugation at 1700× *g* for 6 min, intact chloroplasts settled as sediment to the tube bottom, which was finally resuspended with 1× CIB for further use.

### 4.6. Stress Treatments

For the salt and rose bengal treatments, sterilized seeds were plated on MS medium supplemented with increasing concentrations (25, 50, and 100 mM) of NaCl or 3 µM of rose bengal, incubated at 4 °C for 2 days, and then placed at 22 °C under a 12 h day cycle (80–100 µE/m^2^/s). For high-light treatments, 2-week-old plants, grown under normal conditions, were transferred to high-intensity light (300 µE/m^2^/s) under a long-day condition (16 h light/8 h dark) for 6 days. Photographs were taken on the seventh to fourteenth days after treatments.

### 4.7. Quantification of JA-Ile and (+)-12-oxo-Phytodienoic Acid

The samples of (+)-7-*iso*-jasmonoyl-L-isoleucine [JA-Ile; *N*-(2-((1*R*,2*R*)-3-oxo-2-(2*Z*)-2-pentenyl-cyclopentaneacetic acid)-L-isoleucine) and (+)-12-oxo-phytodienoic acid [OPDA; (1*S*,2*S*)-3-oxo-2-(2*Z*-pentenyl)-cyclopent-4-ene-1-octanoic acid] were prepared from leaves, harvested at 0 and 3 h after wounding. The jasmonates were separated by a Vanquish UHPLC system (Thermo Scientific) equipped with a reversed-phased column (C18 Luna 5.0 μm, 150 × 2.1 mm; Phenomenex) by using a binary solvent system composed of water with 0.1% (*v*/*v*) HCOOH and MeOH with 0.1% (*v*/*v*) HCOOH as a mobile phase at a flow rate of 0.2 mL/min. Separations were performed stepwise: 30% (*v*/*v*) methanol for 4 min, 60% (*v*/*v*) for 6 min, linear increase to 95% for (*v*/*v*) 12.5 min. Identity of jasmonates was confirmed by ion fragmentation on an Orbitrap Exploris 120 mass spectrometer (Thermo scientific, Waltham, MA, USA) with direct injection and operated with a source voltage of 4.0 kV and source temperature of 300 °C. The analysis parameters were optimized by infusing 10 ng/μL of standard compound, (−)-dihydrojasmonic acid [HJA; 2-((1*R*,2*R*)-3-oxo-2-pentylcyclopentyl)acetic acid], JA-Ile and OPDA in 50% (*v*/*v*) MeOH with 0.1% (*v*/*v*) HCOOH at a flow rate of 0.2 mL/min, in multiple reaction-monitoring mode; the fragments *m*/*z* 322.3 → 130.2 (JA-Ile), 213.20 → 195.1 (HJA), and 293.4 → 275.4 (OPDA) were monitored in a positive mode and used for the quantification, respectively.

### 4.8. Photosynthesis Measurements

The data collection for chlorophyll fluorescence-based photosynthetic traits, the total non-photochemical exciton quenching (NPQt) [39], and the quantum efficiency for photosystem II photochemistry (ΦII) [40] was carried out hourly from 9:00 am to 3:00 pm on two leaves of five plants using the hand-held MultispeQ device [40] and uploaded to the PhotosynQ platform “http://www.photosynq.org (accessed on 3 October 2023)” according to the manufacturer’s instructions.

### 4.9. Quantification of GSH and GSSG

The levels of GSH and GSSG present in leaf tissues were measured by using a GSH (GSH/GSSG/total) fluorometric assay kit (BioVision, MA, USA) according to the manufacturer’s instructions. Briefly, leaf tissues were homogenized in ice cold 0.1 M phosphate buffer, PH 7.4, containing 5 mM EDTA, and subsequently treated with 6 N perchloric acid (PCA) to extract GSH and GSSG. PCA was then precipitated by 6 N KOH immediately before assays. For GSH detections, a fluorescence probe o-phthalaldehyde (OPA) was directly added to KOH-neutralized samples, whereas—for GSSG detections—OPA was added to the samples after pre-incubating with 1-methyl-2-vinylpuridinium triflate and GSH reductase to quench pre-existed GSH, and next convert GSSG to GSH, respectively. Finally, levels of GSHs labeled with OPA were fluorometrically measured at Ex/Em = 340/420 nm using the BioTek reader (Agilent, CA, USA).

### 4.10. Semiquantitative and Quantitative RT-PCR

Total leaf RNA was prepared using TRIzol reagent (Invitrogen, Waltham, MA, USA) and the Direct-zol RNA Kit (Zymo Research, Irvine, CA, USA) according to the manufacturer’s instructions. RNA qualities were assessed by agarose gel electrophoresis and NanoDrop (*A_260_*/*A_280_* > 1.8 and *A_260_*/*A_230_* > 2.0) [66]. RT reactions were performed using an oligo(dT) reverse primer and a qScript reverse transcriptase (Quantabio, Beverly, MA, USA). Semi-quantitative RT-PCR was then performed using 2 μL cDNA with *Taq* 2× master mix (New England BioLabs, Ipswich, MA, USA) at an annealing temperature of 55 °C for primer pairs (Table S1) for 30 cycles, whereas quantitative PCR was performed with the PerfeCT^®^ SYBR^®^ Green Fast Mix^®^ Reaction Mixes (QuantaBio, MA, USA) in the CFX96 Touch^TM^ (Bio-Rad, Hercules, CA, USA) PCR system cycled 40 times using gene-specific primer sets (Table S6). The annealing temperature for the primer pairs was 53 °C. To determine the relative abundance of target transcripts, the sample cDNA was assessed with housekeeping genes, *POLYUBIQUITIN* (*UBC*) [38], and the average threshold cycle (i.e., Ct) was normalized to that of *UBC* as 2^−ΔCt^ where −ΔCt = (Ct,_gene_ − Ct,_UBC_).

### 4.11. Statistical Analysis

All statistical analyses were performed using R statistics software v4.3.1 (R Foundation for Statistical Computing).

## Data Availability

The data and results presented in this study are available on request from the first author and corresponding author.

## Supplementary Materials

The following supporting information can be downloaded at: https://www.mdpi.com/article/10.3390/plants12213745/s1, Figure S1: S-glutathionylation modulates the quaternary structure of 2CPA; Figure S2: Temporal changes in the level accumulations of GSSG, OPDA and JA-Ile in wounded WT (Col-0) and mutant (*jar1*, *cyp20-3* and *∆2cp*) plants; Figure S3: CYP20-3/OPDA signaling stimulates the stress-responsive accumulations of GSH; Figure S4: A potential crosstalk between CYP20-3-dependent OPDA signaling and ROS signaling; Table S1: Theoretical masses used to identify the oxidized and glutathionylated forms of 2CPA; Table S2: The results of statistical analyses by Tukey-Kramer honestly significant difference test on all pairs for Figure 4B, Figures S2A and S3; Table S3: The results of statistical analyses by Tukey-Kramer honestly significant difference test on all pairs for Figure 4D; Table S4: The results of statistical analyses by Tukey-Kramer honestly significant difference test on all pairs for Figure 4E,F; Table S5: The results of statistical analyses by Tukey-Kramer honestly significant difference test on all pairs for Figure S4B; Table S6: Oligonucleotides used in this study.

## Abbreviations

Antibody (α); ascorbate peroxidase (APX); β-mercaptoethanol (β-mer); cyclophilin 20-3 (CYP20-3); 2-cysteine peroxiredoxin A (2CPA); cysteine (Cys); Cys synthase complex (CSC); dihydrojasmonic acid (HJA); disulfide form glutathione (GSSG); *N*-ehylmaleimide (NEM); electrons (e^−^, H^+^); electron transport chain (ETC); glutathione (GSH); glutathione S-transferase (GST); glutaredoxin (GRX); heat shock protein (HSP); immunoblot (IB); jasmonic acid (JA); jasmonoyl-isoleucine (JA-Ile); oxo-phytodienoic acid (OPDA); OPDA-responsive genes (ORGs); peptidyl-prolyl *cis*-*trans* isomerase (PPIase); photosystem (PS); PSII quantum yield (Φ_II_); polyubiquitin (UBC); protein sulfhydryl groups (PSH); reactive electrophilic species (RES); reactive oxygen species (ROS); serine (Ser); serine acetyltransferase (SAT); thioredoxin (TRX); total nonphotochemical quenching (NPQ_(T)_); tris(2-carboxyethyl)phosphine hydrochloride (TCEP-HCl); water-water cycle (WWC).
