# Supplementary material for: Reduced GSH Acts as a Metabolic Cue of OPDA Signaling in Coregulating Photosynthesis and Defense Activation under Stress
Source: Plants (Basel). 2023 Nov 1;12(21):3745. doi: 10.3390/plants12213745 (PMC10648297; doi:10.3390/plants12213745)
Supplement: Supplementary file 1 [file plants-12-03745-s001.zip › plants-2662203-supplementary.pdf]

Supplementary Information for:

Reduced GSH acts as a metabolic cue of OPDA signaling in coregulating photosynthesis and defense activation under stress

Ashna Adhikari and Sang-Wook Park

Department of Entomology and Plant Pathology, Auburn University, Auburn, AL 36849

CONTENTS

Fig. S1. S-glutathionylation modulates the quaternary structure of 2CPA.

Fig. S2. Temporal changes in the level accumulations of GSSG, OPDA and JA-Ile in wounded WT (Col-0) and mutant (jar1, cyp20-3 and Δ*2cp*) plants.

Fig. S3. CYP20-3/OPDA signaling stimulates the stress-responsive accumulations of GSH.

Fig. S4. A potential crosstalk between CYP20-3-dependent OPDA signaling and ROS signaling.

Table S1. Theoretical masses used to identify the oxidized and glutathionylated forms of 2CPA

Table S2. The results of statistical analyses by Tukey-Kramer honestly significant difference test on all pairs for Figs 4B, S2A and S3.

Table S3. The results of statistical analyses by Tukey-Kramer honestly significant difference test on all pairs for Figs 4D.

Table S4. The results of statistical analyses by Tukey-Kramer honestly significant difference test on all pairs for Figs 4E and 4F.

Table S5. The results of statistical analyses by Tukey-Kramer honestly significant difference test on all pairs for Figs S4B.

Table S6. Oligonucleotides used in this study.

SUPLLEMENTAL REFERENCES

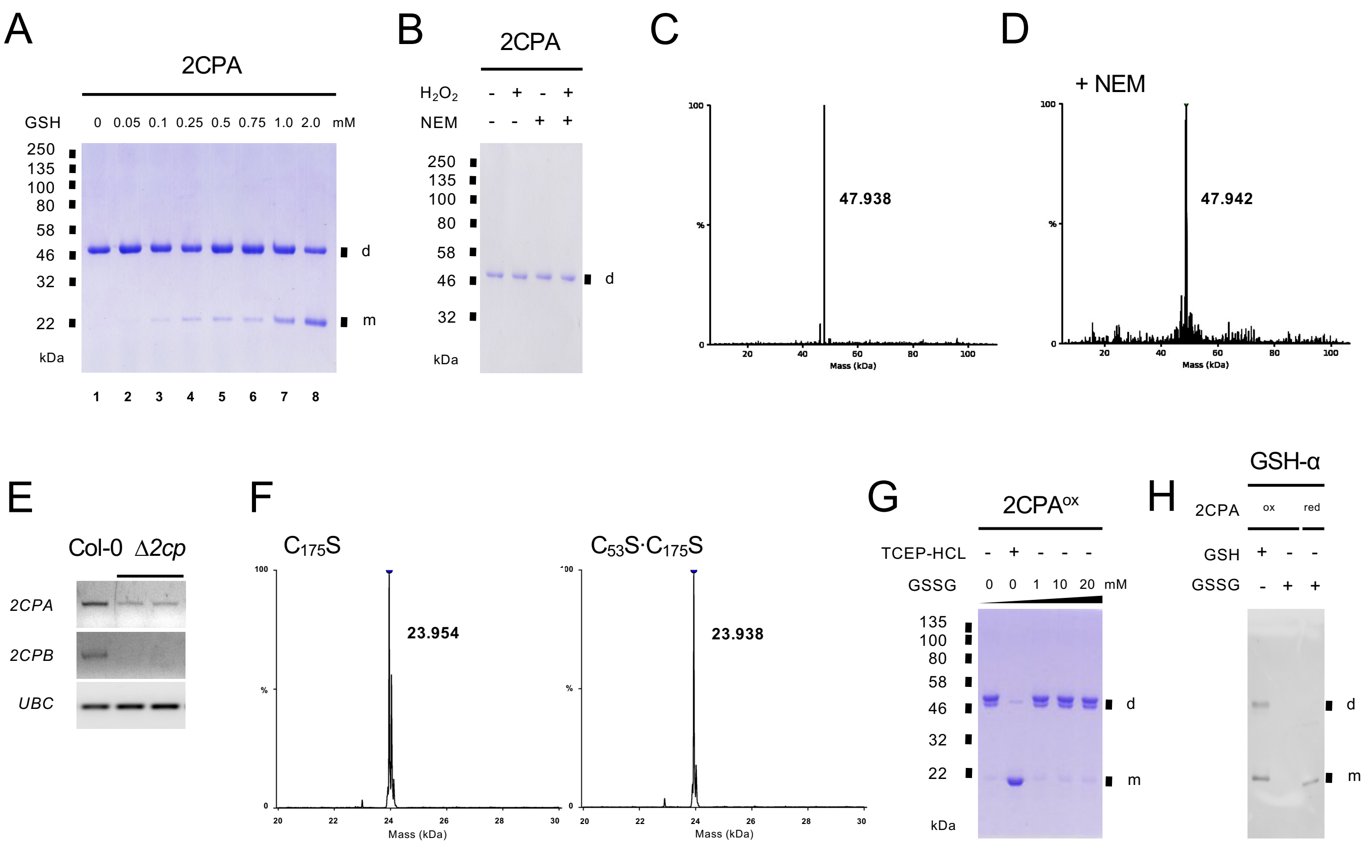

Figure S1. S-glutathionylation modulates the quaternary structure of 2CPA. (A) Reduced GSH stimulates the monomerization of 2CPA in a concentration-dependent manner. Homodimeric oxidized 2CPAs$^{(ox)}$ (lane 1) were subjected to nonreducing SDS/PAGE, following 30-min incubation with the increasing concentration of GHS (0 to 2 mM) at 25 ^{o}C. **(B-D)** In our experimental buffer (50 mM Tris-HCl, pH 7.5) condition, 2CPA forms an obligatory homodimer through double disulfide bonds. 2CPAs (~23.975 kDa), in the presence or absence of 10 μM H_2O_2 and/or 30 mM NEM (0.125 kDa), were subjected to nonreducing SDS/PAGE **(B)** or LC/MS **(C, D)** exhibiting that ***i***) two 2CPAs assemble a dimeric structure (~47.950 kDa, **Supplemental Table S1**), and ***ii***) NEM did not derivatize a dimeric 2CPA. The result elucidates that a homodimeric 2CPA forms double disulfide (S-S) bonds. **(E)** The Δ*2cp* attenuates the level expression of *2CPA*. Semiquantitative RT-PCR analyses of *2CPA* and *2CPB* in WT (Col-0) and Δ*2cp* mutant plants. Total RNAs were prepared from the leaves of each plant, and transcript levels of *UBC* (1) were used as an equal loading control. **(F)** GSH does not S-glutathionylates $C_{175}S$ and $C_{53}S{\cdot}C_{175}S$ mutant 2CPAs. A single or double Cys to Ser mutant 2CPAs ($C_{175}S$ and $C_{53}S{\cdot}C_{175}S$, 1 μM) were incubated with 2 mM GSH for 30 min, and analyzed by LC/MS. **(G, H)** GSSG is not able S-glutathionylate dimeric 2CPA^{ox}. **(G)** The 1 μM 2CPA^{ox} incubated with/without various concentrations (0, 1, 10 and 20 mM) of GSSG, or 5 mM TCEP-HCL for 60 min following 30 mM NEM for 30 min, were subjected to nonreducing SDS/PAGE. **(H)** The 1 μM 2CPA^{ox} or 2CPAs^{red}, incubated with 1 mM GSH or 20 mM GSSG, subjected to nonreducing SDS/GAPGE and IB analyzed using GSH-α. **(A, B, G, H)** Gel was stained with Coomassie Brilliant Blue, and the standard MW sizes (kDa) were indicated in the left of gels. d, dimeric 2CPA. m, monomeric 2CPA. Note that all proteins used in this study were tag-free versions.

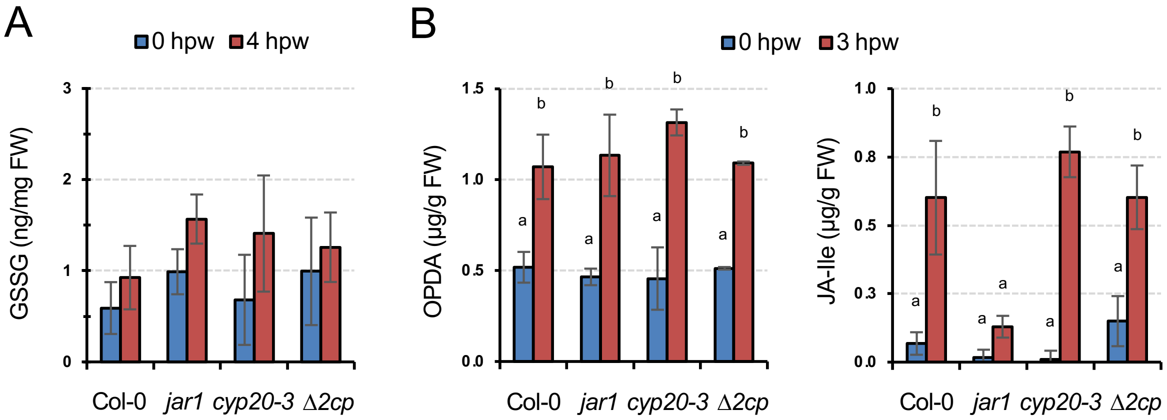

Figure S2. Temporal changes in the level accumulations of GSSG, OPDA and JA-Ile in wounded WT (Col-0) and mutant (*jar1*, *cyp20-3* and Δ*2cp*) plants. **(A)** Glutathione was extracted at 0 and 4 hpw (mean ± SD; 3 independent experiments, n = 3), were measured by the GSH/GSSG/total fluorometric assay kit (BioVision), according to the manufacturer's instruction. The results of statistical analyses are summarized in **Supplemental Table S2**. **(B)** Jasmonates were extracted from leaves at 0 and 3 hpw (mean ± SD; n = 3). Different letters indicate statistically significant differences between genotypes (Tukey–Kramer honestly significant difference test on all pairs; α = 0.05).

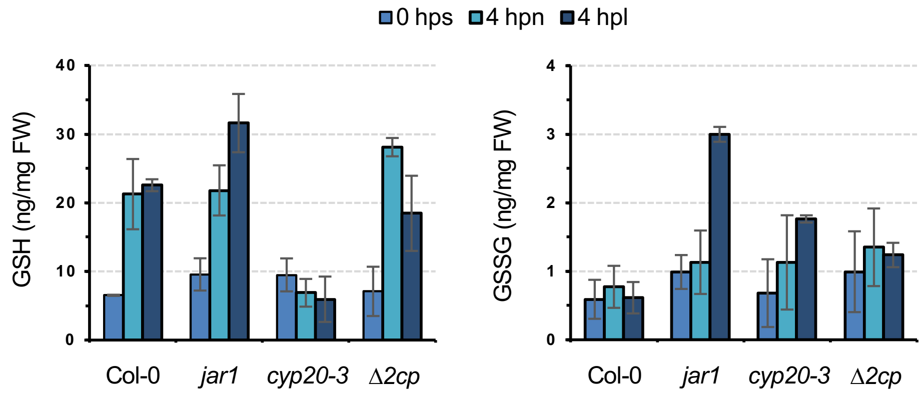

Figure S3. CYP20-3/OPDA signaling stimulates the stress-responsive accumulations of GSH. Levels of GSH and GSSG in stressed WT and mutant (*jar1*, *cyp20-3* and Δ*2cp*) plant leaves, extracted at 0 hps (hr-post-stress), 4 hpn (hr-post-NaCl treat) and 4 hpl (hr-post-excess light treat), were measured (mean ± SD; n = 3) by a GSH (GSH/GSSG/total) fluorometric assay kit (BioVision), according to the manufacturer's instruction. The results of statistical analyses are summarized in **Supplemental Table S2.**

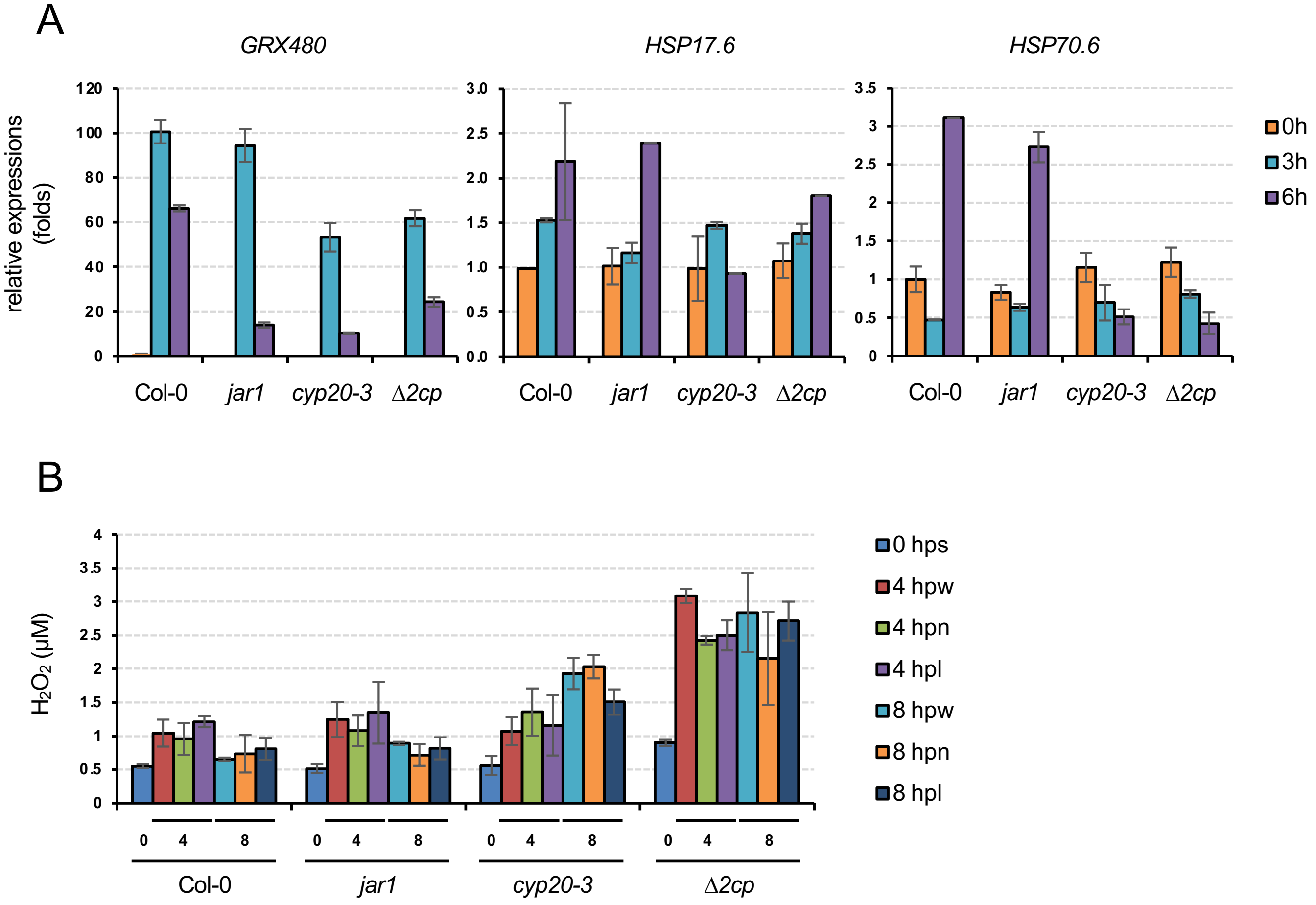

Figure S4. A potential crosstalk between CYP20-3-dependent OPDA signaling and ROS signaling. **(A)** *cyp20-3* and Δ*2cp* mutant plants attenuate the level expression of selective ORGs. Time-resolved quantitative RT-PCR analyses in wounded WT (Col-0) and mutant (*jar1*, *cyp20-3* and Δ*2cp*) plants demonstrated that the transcriptional induction of selective ORGs, particularly those involved in general defense responses such as *GLUTAREDOXIN 480* (*GRX480*), *HEAT SHOCK PROTEIN 17.6* (*HSP17.6*) and *HSP70* (2, 3, 4) was impaired not only in wounded *cyp20-3*, but also wounded Δ*2cp*. Wounded Δ*2cp* however operates, unlike *cyp20-3*, intact OPDA and reductant signaling (**Figure 4B, 4G**) whereas both Δ*2cp* and *cyp20-3* are incapable of balancing photosynthetic H_2O_2 (**Figure 4D; Supplemental Table S3**), potentially underpinning a cellular network that intertwines reductant and oxidative (ROS) signaling in controlling plant environmental plasticity under different ecological conditions. Total RNAs were prepared from the leaves of each plant, and transcript levels of *UBC* (5) were used as an equal loading control. **(B)** CYP20-3-dependent OPDA signaling fosters the peroxidase activity of 2CPA during plant stress responses. Cellular levels of H_2O_2 in stressed WT and mutant (*jar1*, *cyp20-3* and Δ*2cp*) plant leaves were measured (mean ± SD; *n* = 9) at 0 hps, 4 and 8 hpw, 4 and 8 hpn, and 4 and 8 hpl by the eFOX assay method, as described previously (1, 6). The results of statistical analyses are summarized in **Supplemental Table S5.**

Table S1. Theoretical masses used to identify the oxidized and glutathionylated forms of 2CPA.

Species	Molecular mass[a] (*kDa*)
Reduced monomer	24.225
Monomer with single glutathionylation	24.407
Monomer with double glutathionylation	24.589
Dimer	47.950
Dimer with single glutathionylation	48.382
Dimer with double glutathionylation	48.564

[a] Based on complete alkylation of all cysteines, namely Cys-53 (C_R) and Cys-175 (C_P) in the reduced WT protein with NEM (+ 125 Da). Molecular mass of non-alkylated reduced monomer of 2CPA is 23,975 Da.

Table S2. Statistical analysis (Tukey-Kramer test) on the level of GSH and GSSG in stressed WT (Col-0) and mutant (*jar1*, *cyp20-3* and Δ*2cp*) plant leaves at 0 hpw/s (hr-poststress), 4 hpw (hr-postwounding), 4 hpn (hr-post-NaCl treat) and 4 hpl (hr-post-excess light treat) (mean ± SD; 3 independent experiments, *n* = 3) in **Figure 4B; Supplemental Figure S2A, S3.**

genotypes	hps	GSH	GSSG
Col-0	0 hps	cde	a
	4 hpw	ab	a
	4 hpn	ab	a
	4 hpl	ab	a
jar1	0 hps	cd	a
	4 hpw	ab	ab
	4 hpn	ab	ab
	4 hpl	a	ab
cyp20-3	0 hps	cd	a
	4 hpw	cde	a
	4 hpn	cde	a
	4 hpl	cde	ab
Δ2cp	0 hps	d	a
	4 hpw	ab	a
	4 hpn	a	ab
	4 hpl	ab	a

Table S3. Statistical analysis (Tukey-Kramer test) on the level of H_2O_2 in wounded WT (Col-0) and mutant (*jar1*, *cyp20-3* and Δ*2cp*) plant leaves at 0, 4, 8 and 12 hpw (mean ± SD; 3 independent experiments, *n* = 3) in **Figure 4D.**

hpw	genotypes	letter	genotypes	letter
0 hpw	WT (Col-0)	ab	*cyp20-3*	ab
4 hpw		ab		ab
8 hpw		ab		bc
12 hpw		ab		c
0 hpw	*jar1*	ab	*Δ2cp*	b
4 hpw		ab		c
8 hpw		ab		c
12 hpw		ab		c

Table S4. Statistical analysis (Tukey-Kramer test) on the photosynthetic efficacy (total nonphotochemical quenching [NPQt]) and the photosynthetic efficacy (PSII quantum yield [Φ_{II}]) of unwounded (-) and wounded (+) WT (Col-0) and mutant (*jar1*, *cyp20-3* and Δ*2cp*) plant leaves at 0, 1, 2, 3, 4, 5 and 6 hpw (mean ± SD; 3 independent experiments, *n* = 9) in **Figure 4E, 4F.**

hpw	genotypes	NPQt		Φ_{II}	
		-wound	+wound	-wound	+wound
0 hpw	Col-0	ab	ij	a	ef
1 hpw		abcd	ab	a	abcdef
2 hpw		abcd	ab	a	abcdef
3 hpw		ab	abcdefg	a	bcef
4 hpw		ab	bcdefgh	a	cdef
5 hpw		ab	cdefgh	a	def
6 hpw		abcd	defghi	a	cdef
0 hpw	*jar1*	a	j	b	a
1 hpw		abcd	ghij	a	ef
2 hpw		abcd	ghij	a	bcdef
3 hpw		abcd	ghij	a	abcdef
4 hpw		abcd	ghij	a	bcdef
5 hpw		abcd	hij	a	ef
6 hpw		abcd	fghi	a	f
0 hpw	*cyp20-3*	bcd	efghi	a	ef
1 hpw		bcd	bcdefgh	a	cdef
2 hpw		abcd	abcde	a	abcdef
3 hpw		abcd	abcd	a	abcdef
4 hpw		abc	abcd	a	abcdef
5 hpw		abcd	abc	a	abcde
6 hpw		abcd	a	a	abcd
0 hpw	*Δ2cp*	abcd	ghij	a	f
1 hpw		abcd	abcd	a	cdef
2 hpw		bcd	abcdef	a	cdef
3 hpw		d	abcd	a	cdef
4 hpw		abcd	a	a	abc
5 hpw		abcd	a	a	a
6 hpw		cd	a	a	ab

Table S5. Statistical analysis (Tukey-Kramer test) on the level of H_2O_2 in stressed WT (Col-0) and mutant (*jar1*, *cyp20-3* and Δ*2cp*) plant leaves at 0 hps, 4 and 8 hpw, 4 and 8 hpn and 4 and 8 hpl (mean ± SD; 3 independent experiments, n = 9) in **Supplemental Figure S4B.**

genotypes	hps	letter (4 hps)	letter (8 hps)
Col-0	0 hps	st	
	hpw	klmno	opq
	hpn	lmnopq	mnop
	hpl	ijklmn	lmno
jar1	0 hps	t	
	hpw	hijklm	klmno
	hpn	jklmno	nopq
	hpl	ghijk	lmno
cyp20-3	0 hps	st	
	hpw	klmno	def
	hpn	ghijk	cdef
	hpl	efghi	fghij
Δ2cp	0 hps	mnopqr	
	hpw	a	ab
	hpn	abcd	bcde
	hpl	abcd	abc

Table S6. Oligonucleotides used in this study.

Name	Direction	Sequence, 5′ to 3′	purpose
2CPA·C_{53}S·F†	Forward	TTGGACTTTACTTTCGTCAGCCCAACAGAGATTACTG	Mutagenesis††
2CPA·C_{53}S·R†	Reverse	CAGTAATCTCTGTTGGGCTGACGAAAGTAAAGTCCAA	Mutagenesis††
2CPA·C_{175}S·F	Forward	CCCGGATGAAGTCAGCCCAGCAGGATG	Mutagenesis††
2CPA·C_{175}S·R	Reverse	CATCCTGCTGGGCTGACTTCATCCGGG	Mutagenesis††
2CPA·Fw	Forward	AACTACTCTCATCTCTTCTCC	RT-PCR
2CPA·Rev	Reverse	AGGGAAGAGCGTCGAGCAAAT	RT-PCR
2CPB·Fw	Forward	TTCTTCCACCACCCTACT	RT-PCR
2CPB·Rev	Reverse	GGAACCGAGACTGGAGAAC	RT-PCR
CYP18D11·Fw	Forward	TCTCAACATG^GGTTTGTGAA	qRT-PCR^
CYP18D11·Rev	Reverse	AAGTATC^ATAACAAGTATGA	qRT-PCR^
HSP17.6·Fw	Forward	CTTGCCTGGATTAAGAAGG	qRT-PCR
HSP17.6·Rev	Reverse	CATCGCAGCCTTAACCTGAT	qRT-PCR
HSP70·Fw	Forward	GGAAAGTTCGAGCTCAGTGG	qRT-PCR
HSP70·Rev	Reverse	ACCTTCCCTTGTCGTTTGTG	qRT-PCR
GST6·Fw	Forward	TGCCCTCAACCCCTTCGGTC	qRT-PCR
GST6·Rev	Reverse	GGTTGCCTTGACTTTCTTGC	qRT-PCR
GST8·Fw	Forward	TGACAAGAAGCTG^TATGATGC	qRT-PCR^
GST8·Rev	Reverse	ACATAGCCAAAGTCATCGCC	qRT-PCR
GRX480·Fw	Forward	TGATTGTGATTGGACGGAGA	qRT-PCR
GRX480·Rev	Reverse	TAAACCGCCGGTAACTTCAC	qRT-PCR
UBC·Fw	Forward	CTGCGACTCAG^GGAATCTTCTAA	qRT-PCR^
UBC·Rev	Reverse	TTGTGCCATTGAATTGAACCC	qRT-PCR

† Primers, 2CPA·C_{53}S·F and 2CPA·C_{53}S·R, were used to generate the 2CPAC_{53}S·C_{175}S plasmid using the 2CPAC_{175}S plasmid as the template.

†† Mutated nucleotide bases are grey highlighted.

^ Position of an exon-exon junction.

SUPPLEMENTAL REFERENCES

(1) Cheeseman, J.M. (2006). Hydrogen peroxide concentrations in leaves under natural conditions. *J. Exp. Bot.* **57**, 2435-2444.

(2) Mou, Z., Fan, W., and Dong, X. (2003). Inducers of plant systemic acquired resistance regulate NPR1 function through redox changes. *Cell* **113**, 935-944.

(3) Finka, A., Mattoo, R.U., and Golobinoff, P (2011). Meta-analysis of heat- and chemically upregulated chaperone genes in plant and human cells. *Cell Stress Chaperones* **16**, 15-31.

(4) Park, S.W., Li, W., Viehhauser, A., He, B., Kim, S., Nilsson, A.K., Andersson, M.X., Kittle, J.D., Ambavaram, M.M., Luan, S. et al. (2013). Cyclophilin 20-3 relays a 12-*oxo*-phytodienoic acid signal during stress responsive regulation of cellular redox homeostasis. *Proc. Natl. Acad. Sci.* USA **110**, 9559-9564.

(5) Czechowski, T., Stitt, M., Altmann, T., Udvardi, M.K., and Scheible, W.R. (2005). Genome-wide identification and testing of superior reference genes for transcript normalization in *Arabidopsis*. *Plant Physiol.* **139**, 5-17.

(6) Liu, W., Dos Santos, I.B., Moye, A., and Park, S.W. (2020). CYP20-3 deglutathionylates 2-CysPRX A and suppresses peroxide detoxification during heat stress. *Life Sci. Alliance* **3**, e202000775.
